# Health-income inequality: the effects of the Icelandic economic collapse

**DOI:** 10.1186/1475-9276-13-50

**Published:** 2014-07-25

**Authors:** Tinna Laufey Ásgeirsdóttir, Dagný Ósk Ragnarsdóttir

**Affiliations:** 1 Department of Economics, University of Iceland, Oddi v/Sturlugötu, 101 Reykjavík, Iceland

**Keywords:** Concentration index, Decomposition, Health-income inequalities, Economic conditions, Crisis, Iceland

## Abstract

**Introduction:**

Health-income inequality has been the focus of many studies. The relationship between economic conditions and health has also been widely studied. However, not much is known about how changes in aggregate economic conditions relate to health-income inequality. Nevertheless, such knowledge would have both scientific and practical value as substantial public expenditures are used to decrease such inequalities and opportunities to do so may differ over the business cycle. For this reason we examine the effect of the Icelandic economic collapse in 2008 on health-income inequality.

**Methods:**

The data used come from a health and lifestyle survey carried out by the Public Health Institute of Iceland in 2007 and 2009. A stratified random sample of 9,807 individuals 18–79 years old received questionnaires and a total of 42.1% answered in both years. As measures of health-income inequality, health-income concentration indices are calculated and decomposed into individual-level determinants. Self-assessed health is used as the health measure in the analyses, but three different measures of income are used: individual income, household income, and equivalized household income.

**Results:**

In both years there is evidence of health-income inequality favoring the better off. However, changes are apparent between years. For males health-income inequality increases after the crisis while it remains fairly stable for females or slightly decreases. The decomposition analyses show that income itself and disability constitute the most substantial determinants of inequality. The largest increases in contributions between years for males come from being a student, having low education and being obese, as well as age and income but those changes are sensitive to the income measure used.

**Conclusions:**

Changes in health and income over the business cycle can differ across socioeconomic strata, resulting in cyclicality of income-related health distributions. As substantial fiscal expenditures go to limiting the relationship between income and health, the business-cycle effect on equality, which has up until now not received much attention, needs to be considered.

## Introduction

The reasons for government involvement in health care and prevention are multiple, but the reduction of income-related health inequality is a partial motivation. A mitigation of the health-income relationship has been the focus of large-scale and growing government expenditures in many countries. The scale of the expenditures involved, makes it an important subject to examine. In this paper we focus on the relationship between fluctuations in aggregate economic activity, that is business cycles, and income-related health inequality. This paper thus relates to two strands of literature that need to be mentioned, those of inequalities in health on one hand and business cycles and health on the other hand. How those relate to one another is however not clear and has received limited attention.

An overwhelming majority of studies indicate that high-income individuals possess better health than their low-income counterparts, although the causality of this relationship is still somewhat convoluted [1]. We know that aggregate cyclical changes in economic activity have an effect on health (see for example [2]) and by definition, also on income, which in turn could result in changes in health-income inequality. However, to date examination of the relationship between business cycles and income-related health is in its infancy.

Health-income inequality, without regard for the business cycle, has been studied across countries using different methods, including the concentration index. Van Doorslaer et al. [3] used concentration indices to examine income-related inequalities in health for nine countries. Individuals were ranked by equivalent household income and health was measured by self-assessed health (SAH). The study found inequalities in health favoring high-income individuals in all nine countries, but with only nine countries, it was impossible to draw conclusions about the relationship between the level of inequality and aggregate economic conditions in those countries.

More studies have emerged that compare socioeconomic inequality in health across countries using similar methods to those we employ. Van Doorslaer and Koolman [4] studied health-income inequality in thirteen European countries and found health-income inequalities favoring high-income individuals in all of them, particularly sharp ones in Portugal, but also in the UK and Denmark, whereas they were low in the Netherlands and Germany, and also in Italy, Belgium, Spain, Austria and Ireland. Thus results did not show a clear pattern with regard to the economic conditions in those countries. Ásgeirsdóttir and Ragnarsdóttir [5] calculated concentration indices for 26 European countries, which allowed for some cross-country examination with regard to economic, cultural and institutional settings. Business cycle effects were not a part of their analyses, but they examined cross-country patterns with respect to Gross Domestic Product (GDP) and found no apparent relationship between income-related health inequality and GDP. However, short-term fluctuations in GDP, such as business cycles, can have different effects than the general GDP level of a country. From the literature on health over the business cycle, we do, for example, know that while high GDP generally leads to better health, short-term fluctuations in GDP can have a different effect on health.

Ruhm [2], for example, found temporary downturns in the United States over the period 1972–1991 to reduce eight of ten sources of fatalities he examined as well as total mortality. Similar results, indicating improved health in economic downturns, have been found in studies from Germany, Spain, Sweden, 23 OECD countries, and 8 Pacific Asian countries [6-10]. It should, however, be noted that other recent research has found evidence of the opposite. Economou, Nikolaou and Theodossiou [11] examined the effects of national unemployment rates on mortality in 13 European Union countries and found a strong positive relationship between economic downturns and mortality rates. A counter-cyclical fluctuation of mortality has also been found in Sweden. Using individual level data Gerdtham and Johannesson [12] found mortality risk for males to be higher in downturns, but no effect was found for females. Stuckler et al. [13] found no consistent evidence that economic conditions affected all-cause mortality in 26 European Union countries in the years 1970–2007, but they found mortality to be more sensitive to economic downturns in some countries with weaker social systems.

The purpose of this study is to shed light on the possible interplay between the two above-mentioned literatures. As the business cycle constitutes fluctuations in economic activity over time, a logical first step would be to consider trends in income-related inequalities in health over time, which have been examined to some extent. Socioeconomic inequalities in health in ten European countries in the 1980s and 1990s were found to be quite stable over time, despite some variation in trends across countries [14]. In another study, a fairly consistent increase over the same time period was found in the Netherlands [15]. Only a few studies have focused on what factors influence changes in health-income inequality over time, as is the focus in this paper with respect to business cycles. A few can, however, be mentioned. An increase in income-related health inequality was found in the UK over the years 1979–1995, mainly caused by increases in income inequality and average income [16]. In England, the contributions of smoking and obesity to income-related health inequality in 1998–2006 were examined, but were only found to make a modest contribution [17]. A Japanese study examined what contributing factors influenced changes in health-income inequality in Japan in 1986–2007. They found inequalities in health to decrease after the Asian economic crisis in the late 1990s, mainly due to a decrease in the contribution of income [18]. A decrease in income-related health inequalities following an economic crisis has also been observed in Finland [19], as well as in another Japanese study [20] where there was a decrease in inequality among older working-age people after the crisis. However, there was an increase in the health-income gradient among younger working-age people.

In this paper we examine how the Icelandic economic collapse of 2008 affected the income-related distribution of health. The Icelandic economic collapse has already proved itself to be a valuable identification tool [21-25]. This is partly due to its sharpness that creates a clear distinction between the pre-collapse bubble and the post-collapse recession. (The reader is referred to the previous literature for greater details on the Icelandic economic collapse in general: [26], or specifically as a treatment: [24]). As the research field has struggled methodologically with the treatment of trends, such a clear before and after picture, coupled with valuable data, is of great importance. We take advantage of this. Concentration indices will be calculated for self-assessed health in 2007 and 2009, incidentally a year before and after the dramatic collapse. Both gender specific as well as overall indices are calculated and comparisons between years made. Subsequently, the concentration indices will be decomposed and contributions of each determinant to inequality in health calculated. The decomposition is important as it sheds light on whether those interested in decreasing health variations are likely to find effective ways to do so through different channels at various stages of the business cycle.

### Data and methods

The data utilized in this study come from a health and lifestyle survey carried out by the Public Health Institute of Iceland, now the Directorate of Health, in 2007 and 2009. The survey contains questions on health and lifestyle, as well as work related issues and demographics. A stratified random sample of 9,711 citizens 18–79 years old received a questionnaire in 2007. 42.1% of the original sample answered both waves. Matching averages between key variables from the sample and Statistics Iceland showed that the sample is representative of the population when sample weights are used. However, due to deliberate over sampling in some strata, the sample consists of relatively older individuals than the census and relatively more people living outside the capital region. Sample weights are thus included in the analysis to make the sample nationally representative. The health and income variables used will now be discussed.

A four-level self-assessed health variable, ranging from “very good” to “poor” is used. The literature shows that SAH is seen as a good predictor of mortality and morbidity, even in the presence of additional controls [27-37]. Furthermore, this measurement is frequently used and thus current estimates can be held up against comparable health concentration indices for other countries, although it should be kept in mind when comparing results that five levels of health are more common in SAH measures. As the SAH variable has been coded in various ways in the literature on health variations and using it as a continuous four-level variable is somewhat arbitrary, robustness checks were carried out in which the sensitivity to different coding was examined. Further explanations of those estimations and the results they provided are available upon request, but in short they involve using the natural logarithm of health [38,39] as well as dichotomization of health [40]. In all instances, the numeric values of the SAH variable are such that a higher number indicates better health, and thus health concentration indices are calculated, as opposed to the concentration of ill health being measured. Robustness checks showed that the results are qualitatively similar regardless of the health measure used (available upon request).

Three income measures were used in the calculations: average monthly individual income before taxes, average monthly household income before taxes, and average monthly equivalized household income before taxes. Individual income was reported in 10 income groups, while household income was reported in 14 income groups. The midpoint (equidistance from both endpoints) of each group was imputed and the variables used as continuous. The inflation rate between 2007 and 2009 was 27%. To report income in real terms the data from 2007 was multiplied by 1.27, as has been done in other publications using the same data [24]. All income measures were scaled to million ISK. OECD-modified equivalence scale was used to calculate equivalized household income. Equivalized household income measures access to finances through own and family-members income, taking into account economies of scale in household production. It is constructed from household income divided by equivalized household size, which assigns the value 1 to an adult in a household, 0.5 to each additional adult and 0.3 to each child. According to the OECD-modified scale, children are defined as those younger than 14 years of age, but here children are defined as those younger than 18 years old as the questionnaire does not distinguish between the subjects’ children who are younger or older than 14.

Demographics used in the analysis are marital status, education, employment status, age and gender. Furthermore variables on body weight, smoking and alcohol consumption are included. Marital status dummy variables include married (reference category), cohabiting, in a relationship, single, divorced and widowed. The inclusion of those variables is based on theory and previous literature. Inequalities due to age and gender are to a large extent unavoidable, and thus need to be controlled for in order to obtain the more policy-relevant avoidable inequality. Marital status is a basic demographic that is highly related to income, especially household income, which is used in this analysis. Labor-market status changes considerably over the business cycle and could be the cause of changes in income-related health inequality. The business cycle is also known to be related to lifestyle choices that affect health and vary according to income [24]. Therefore body weight, smoking and drinking are included, but those constitute the go-to list of risky behaviors and conditions that affect health.

In 2007 respondents could choose from eight answers on highest level of education but in 2009 two other response options were added. Due to the increased quality in answers between waves, answers from 2009 were used with imputations from 2007 in cases of missing 2009 responses. Three educational groups were constructed. The group *low education* includes those who had finished primary or secondary school, *medium education* includes those who had finished vocational master, journeyman certificate, or high school or equivalent, and *high education* those who had finished technical graduate, undergraduate degree, master’s degree, or a doctoral degree. The educational categories are coded as separate dummy variables. Employment status includes employee (reference category), business owner, student, homemaker, on parental leave, on temporary illness leave, retired, unemployed, and disability and is similarly coded with separate dummy variables. The body mass index (BMI) was used to proxy body composition. BMI is defined as an individual’s body weight in kilograms divided by the square of his or her height in meters. The optimal BMI level lies between 18.5 and 24.9. Individuals with BMI under 18.5 are considered underweight. Individuals with BMI between 25 and 29.9 are considered overweight and those with BMI 30 or more are considered obese [41]. A dummy variable was created for each BMI category and optimal BMI is used as a reference category. A question on smoking contained three different response categories for those who were current smokers, from smoking daily to smoking less than weekly, and two response categories for those who do not smoke, either that they gave up smoking, or that they have never smoked (reference category). In the analysis those categories are thus used as dummy variables. A question on how often an individual consumed five or more alcoholic beverages in the past twelve months is used as continuous.

The methodology used in the analyses is based on the concentration curve and the corresponding concentration index. The concentration index is based on the Lorenz curve, a cumulative frequency curve, which compares the distribution of a specific variable with the uniform distribution that represents equality. The health-income concentration curve is a plot of the cumulative proportion of health against the cumulative proportion of the population ranked by income. As such, it allows for examination of variations in one variable relative to variations in another variable. The income dimension is captured by the ranking of observations by income on the horizontal axis (with the least advantaged furthest to the left). The cumulative proportion of the health variable is then represented on the vertical axis. The concentration curve can be compared with a diagonal line representing a uniform distribution, or perfect equality. The greater the deviation of the concentration curve from this line, the greater is the inequality. Thus the difference between the Lorenz curve and the concentration curve lies in the cumulative desiderata on the vertical axis. In the case of the Lorenz curve this is the same income variable as used to rank individuals on the horizontal axis, but in the case of the concentration curve it is cumulative health, despite individuals being ranked by income on the horizontal axis.

The concentration index is defined as twice the area between the concentration curve and the diagonal line. The concentration index provides a measure of socio-economic inequality in health. It ranges from -1 to 1, with 0 representing perfect equality and -1 and 1 representing total inequality favoring those with low and high income respectively. The concentration index can be represented by the following formula:

$$\mathrm{CI}\;=1-2\int_{0}^{1}L\left(s\right)\mathrm{ds}$$

where *L(s)* is the cumulative distribution of health, as a function of cumulative income, *s*, and can be computed straightforwardly with individual-level data using the following formula:

$$\mathrm{CI}\;=\frac{2}{\mathrm{n\mu}}\sum_{i=1}^{n}y_{i}R_{i}-1$$

where *y*_*i*_ (*i* = 1, …, *n*) is the health score of individual *i*, *μ* is the mean level of health and *R*_*i*_ is the relative rank by income of individual *i*[3,4,39].

In this study we calculate concentration indices for the years 2007 and 2009, both overall and for males and females separately, using the three different income measures described above. These concentration indices are then compared and differences between years examined.

The concentration index has a number of advantages as a measure of income inequalities in health. Most importantly, it reflects the experience of the entire population and not just those of two extreme socio-economic groups, as other measures frequently used do. The concentration index does not take into account the fact that demographics play a role in generating inequality. However, these factors can be taken into account, for example, by partitioning the concentration index into avoidable and unavoidable (age-gender) health inequality. We use an approach that allows for the subtraction of unavoidable inequality, while unstandardized concentration indices are still shown in the analysis for completeness. Specifically, we decompose the concentration index into contributions of its various determinants, both unavoidable and avoidable, using the following linear regression model:

$$y_{i}=\alpha +\sum_{k}\beta_{k}x_{\mathrm{ki}}+\epsilon_{i}$$

where *y*_*i*_ is the health measure for individual *i*, *x*_*ki*_ is a health determinant for regressor *k* and individual *i* and *ϵ*_*i*_ is the error term. This decomposition is conducted for all concentration indices in the analyses. Given the relationship between *y*_*i*_ and *x*_*ki*_, the concentration index for *y* can be written as follows:

$$\mathrm{CI}=\sum_{k}\left(\frac{\beta_{k}{\overset{¯}{x}}_{k}}{\mu }\right)CI_{k}+\frac{\mathrm{GC}I_{\epsilon }}{\mu }$$

where *μ* is the mean of y, ${\overset{¯}{x}}_{k}$ is the mean of *x*_*k*_, *CI*_*k*_ is the concentration index for *x*_*k*_ and *GCI*_*ϵ*_ is the generalized concentration index for the error term. CI is thus equal to the sum of all the concentration indices of the *k* regressors, weighted by the elasticity of y with respect to *x*_*k*_. *GCI*_*ϵ*_ is the residual and reflects health inequality not explained by systematic variation across income in the *x*_*k*_[3,4,39]. The data were analyzed with the Stata 11.0 software [42]. The study was approved by the Ethics Review Board of Iceland (07–081 and 09–094).

## Results

As the samples used differ slightly between analyses using individual and household income, summary statistics are provided for both samples. Summary statistics for all variables can be found in Table 1 for males and females, only including individuals who answered questions on individual income and self-assessed health in both years. Table 2 includes summary statistics for both genders, only including individuals who answered questions on household income and self-assessed health in both years. It can be seen that females rate their health slightly worse than males on average. Males rate their health slightly better after the crisis, while females rate their health slightly worse after the crisis. Mean income is higher for males than females in all instances and mean income decreases between years for both genders.

**Table 1:** Summary statistics with individual income

**Variables**	**Males**						**Females**					
	**2007**			**2009**			**2007**			**2009**		
	**Mean**	**SD**	**N**	**Mean**	**SD**	**N**	**Mean**	**SD**	**N**	**Mean**	**SD**	**N**
Individual income	5.497	2.977	1689	4.445	2.430	1689	3.600	2.310	1907	3.133	1.818	1907
Health	3.115	0.759	1689	3.123	0.771	1689	3.053	0.790	1907	3.032	0.769	1907
Age	43.137	15.842	1689	45.194	15.780	1673	43.568	15.814	1907	45.483	15.773	1890
Single	0.167	0.373	1681	0.144	0.351	1673	0.142	0.349	1899	0.115	0.319	1892
In a relationship	0.075	0.263	1681	0.071	0.257	1673	0.061	0.240	1899	0.067	0.250	1892
Cohabiting	0.201	0.401	1681	0.182	0.386	1673	0.213	0.410	1899	0.196	0.397	1892
Married	0.517	0.500	1681	0.550	0.498	1673	0.488	0.500	1899	0.506	0.500	1892
Divorced	0.032	0.175	1681	0.042	0.200	1673	0.059	0.235	1899	0.068	0.253	1892
Widowed	0.011	0.104	1681	0.012	0.109	1673	0.040	0.195	1899	0.048	0.213	1892
Low education	0.215	0.411	1689	0.215	0.411	1689	0.306	0.461	1907	0.306	0.461	1907
Medium education	0.426	0.495	1689	0.426	0.495	1689	0.279	0.449	1907	0.279	0.449	1907
High education	0.359	0.480	1689	0.359	0.480	1689	0.415	0.493	1907	0.415	0.493	1907
Employee	0.820	0.384	1499	0.808	0.394	1311	0.808	0.394	1653	0.797	0.402	1521
Business owner	0.195	0.396	1404	0.230	0.421	1167	0.080	0.271	1512	0.086	0.280	1247
Student	0.208	0.406	1356	0.221	0.415	1048	0.269	0.444	1514	0.256	0.437	1222
Homemaker	0.043	0.202	1355	0.037	0.189	1037	0.162	0.368	1528	0.159	0.365	1249
Parent leave	0.016	0.124	1350	0.019	0.137	1028	0.051	0.219	1473	0.058	0.234	1204
Temporary ill	0.033	0.178	1354	0.031	0.174	1039	0.053	0.224	1478	0.053	0.225	1211
Retired	0.083	0.276	1463	0.128	0.335	1254	0.095	0.293	1607	0.147	0.354	1457
Unemployed	0.026	0.158	1333	0.084	0.278	1040	0.029	0.169	1458	0.068	0.252	1196
Disabled	0.038	0.192	1364	0.050	0.218	1067	0.076	0.265	1510	0.094	0.292	1251
Underweight	0.004	0.060	1689	0.002	0.050	1689	0.007	0.085	1907	0.008	0.092	1907
Optimal	0.332	0.471	1689	0.300	0.458	1689	0.432	0.495	1907	0.412	0.492	1907
Overweight	0.467	0.499	1689	0.499	0.500	1689	0.313	0.464	1907	0.331	0.471	1907
Obese	0.199	0.399	1689	0.200	0.400	1689	0.249	0.432	1907	0.250	0.433	1907
Non-smoker	0.468	0.499	1610	0.468	0.499	1606	0.483	0.500	1823	0.488	0.500	1828
Daily smoker	0.156	0.363	1610	0.114	0.317	1606	0.152	0.359	1823	0.140	0.347	1828
Weekly smoker	0.013	0.114	1610	0.027	0.162	1606	0.023	0.151	1823	0.023	0.150	1828
Seldom smoker	0.029	0.168	1610	0.029	0.169	1606	0.037	0.188	1823	0.020	0.142	1828
Former smoker	0.332	0.471	1610	0.362	0.481	1606	0.304	0.460	1823	0.328	0.470	1828
5+ alcoholic drinks	18.622	34.115	1649	17.255	35.223	1634	6.869	17.817	1856	6.265	20.131	1828

Income is measured in millions of ISK. Health is measured with a four-level self-assessed health variable ranging from 1 to 4. The variable “5+ alcohol drinks” measures how often in the last twelve months the subject consumed five or more alcoholic drinks.

**Table 2:** Summary statistics with household income

**Variables**	**Males**						**Females**					
	**2007**			**2009**			**2007**			**2009**		
	**Mean**	**SD**	**N**	**Mean**	**SD**	**N**	**Mean**	**SD**	**N**	**Mean**	**SD**	**N**
Household income	9.206	5.293	1620	7.568	4.28293	1620	8.150	4.933	1763	6.498	3.851	1763
Equivalized hh income	7.407	4.395	1552	6.151	3.651	1560	6.483	4.054	1707	5.273	3.256	1705
Health	3.123	0.754	1620	3.129	0.766	1620	3.060	0.791	1763	3.039	0.770	1763
Age	42.913	15.652	1620	44.975	15.590	1605	43.005	15.521	1763	44.911	15.482	1748
Single	0.164	0.370	1612	0.140	0.347	1606	0.140	0.347	1758	0.115	0.319	1750
In a relationship	0.072	0.259	1612	0.071	0.257	1606	0.061	0.239	1758	0.068	0.252	1750
Cohabiting	0.205	0.404	1612	0.185	0.388	1606	0.220	0.415	1758	0.202	0.401	1750
Married	0.520	0.500	1612	0.554	0.497	1606	0.485	0.500	1758	0.506	0.500	1750
Divorced	0.031	0.173	1612	0.041	0.197	1606	0.058	0.234	1758	0.067	0.250	1750
Widowed	0.010	0.098	1612	0.010	0.102	1606	0.038	0.191	1758	0.044	0.205	1750
Low education	0.208	0.406	1620	0.208	0.406	1620	0.290	0.454	1763	0.290	0.454	1763
Medium education	0.431	0.495	1620	0.431	0.495	1620	0.285	0.451	1763	0.285	0.451	1763
High education	0.361	0.480	1620	0.361	0.480	1620	0.425	0.495	1763	0.425	0.495	1763
Employee	0.818	0.386	1454	0.810	0.392	1279	0.809	0.394	1551	0.798	0.402	1432
Business owner	0.199	0.399	1362	0.236	0.425	1139	0.082	0.274	1420	0.085	0.278	1181
Student	0.209	0.407	1315	0.216	0.412	1021	0.268	0.443	1425	0.256	0.437	1160
Homemaker	0.041	0.199	1314	0.037	0.188	1009	0.157	0.364	1433	0.157	0.364	1173
Parent leave	0.016	0.126	1309	0.019	0.138	1001	0.051	0.219	1388	0.058	0.234	1141
Temporary ill	0.032	0.175	1313	0.028	0.165	1012	0.050	0.217	1394	0.051	0.220	1146
Retired	0.077	0.267	1406	0.124	0.329	1206	0.084	0.278	1497	0.133	0.340	1348
Unemployed	0.025	0.156	1296	0.080	0.271	1012	0.026	0.159	1375	0.066	0.248	1132
Disabled	0.036	0.187	1320	0.046	0.209	1037	0.072	0.258	1424	0.090	0.286	1186
Underweight	0.002	0.044	1620	0.003	0.050	1620	0.008	0.089	1763	0.009	0.096	1763
Optimal	0.334	0.472	1620	0.298	0.457	1620	0.436	0.496	1763	0.414	0.493	1763
Overweight	0.466	0.499	1620	0.500	0.500	1620	0.309	0.462	1763	0.329	0.470	1763
Obese	0.200	0.400	1620	0.201	0.401	1620	0.248	0.432	1763	0.251	0.433	1763
Non-smoker	0.469	0.499	1556	0.468	0.499	1552	0.476	0.500	1698	0.483	0.500	1702
Daily smoker	0.156	0.363	1556	0.113	0.316	1552	0.156	0.363	1698	0.144	0.351	1702
Weekly smoker	0.014	0.116	1556	0.026	0.160	1552	0.026	0.160	1698	0.024	0.154	1702
Seldom smoker	0.029	0.167	1556	0.030	0.170	1552	0.039	0.193	1698	0.022	0.146	1702
Former smoker	0.332	0.471	1556	0.363	0.481	1552	0.303	0.460	1698	0.328	0.470	1702
5+ alcoholic drinks	18.701	33.683	1588	17.016	33.510	1575	6.750	17.334	1726	6.381	20.469	1697

Income is measured in millions of ISK. Health is measured with a four-level self-assessed health variable ranging from 1 to 4. The variable “5+ alcohol drinks” measures how often in the last twelve months the subject consumed five or more alcoholic drinks.

The interested reader is referred to the Additional file 1 for a fuller description of how the determinants included in the analyses relate to health. The Additional file 1 includes three regression analyses that show the marginal effects of each determinant on health for both years and males and females separately. Additional file 1: Table S1 shows the marginal effects of each determinant on health using individual income as one of the explanatory variables. Additional file 1: Table S2 reports this relationship using household income as one of the explanatory variables and Additional file 1: Table S3 shows results where equivalized household income is one of the explanatory variables.

The male, female and overall concentration indices for the years 2007 and 2009 can be found in Table 3. That table can thus be viewed as presenting the core results of the research project. Income-related health inequality favoring the better off is observed for both genders in both 2007 and 2009. Standardization for age (and gender) has a minimal effect on the concentration indices. The results show that health-income inequality is lower for males than females in all instances. However, inequality for males increased substantially between years using all income measures while there was a slight decrease in inequality between years for females. Due to the larger change for males the overall concentration indices became higher in 2009 than 2007 in all instances. The concentration indices for males show that health-income inequality was highest when household income is the income measure and lowest when individual income is used. For males there is a smaller increase in income-related health inequality between years when the concentration indices are standardized for age. Health-income inequality for females is also highest using household income and lowest using individual income in the calculations. Age standardized concentration indices are slightly lowered in the instances of household income and equivalized household income as the income measures but slightly increase when individual income is the income measure.

**Table 3:** Concentration indices

**Income measure**	**Concentration indices**					
	**Overall**		**Males**		**Females**	
	**2007**	**2009**	**2007**	**2009**	**2007**	**2009**
**Unstandardized CI**						
Individual income	0.0236	0.0249	0.0198	0.0234	0.0275	0.0252
Household Income	0.0312	0.0355	0.0264	0.0347	0.0353	0.0341
Equvalized household income	0.0273	0.0330	0.0211	0.0312	0.0328	0.0325
**Standardized CI**						
Individual income	0.0235	0.0248	0.0192	0.0247	0.0278	0.0252
Household Income	0.0305	0.0332	0.0266	0.0321	0.0342	0.0339
Equvalized household income	0.0270	0.0319	0.0213	0.0297	0.0321	0.0324

The decompositions of the concentration indices for both genders using individual income as the income measure can be found in Table 4. Each column shows percentage contributions from each determinant to income-related health inequality. As described in the methods chapter, the contributions depend on both the elasticity of health with respect to the determinants and the income-related concentration index of each determinant. By far the largest contributions are individual income and disability. Other determinants that contributed considerably to income related health inequality among males come other employment-status variables and from being a daily smoker. However, being a student reduced health inequality quite immensely among males, especially in 2007. For males education also contributed somewhat to inequality, or around 5% for high education in 2007 and low education in 2009. For females, other large contributions to inequality are education, employment status and being obese. This is quite different for males, where both being overweight and obese lowered health inequality by a fair amount in 2007 and slightly in 2009. Those qualitative patterns in contributions to inequality are largely robust to changes in the income measure used. Decomposition results from concentration indices with household income and equivalized household income as the income measures can be seen in Table 5 and Table 6 respectively.

**Table 4:** Decomposition with individual income as the income measure

	**Overall**		**Males**		**Females**	
	**2007**	**2009**	**2007**	**2009**	**2007**	**2009**
	**% contribution**					
**Individual income**	43.67	47.74	64.02	61.77	32.26	25.00
Age	−0.35	−1.57	2.95	−5.58	−0.89	−0.02
Female	0.64	1.80				
Single	3.89	2.71	8.39	12.57	1.87	−0.49
In a relationship	2.08	1.51	−1.15	0.64	2.29	2.75
Cohabiting	0.03	−0.38	0.42	−0.24	0.02	0.26
Divorced	0.12	−0.01	0.29	1.18	−0.03	1.63
Widowed	1.90	0.03	2.10	3.46	0.62	−0.88
Low education	6.12	4.25	0.55	5.49	12.67	4.32
High education	4.70	−1.83	5.38	−0.69	5.04	−1.82
Business owner	−1.84	2.61	−1.64	2.73	−0.35	0.26
Student	−12.47	−10.36	−37.60	−14.63	0.94	−4.97
Homemaker	−0.47	1.09	−0.34	1.45	−3.46	−2.28
Parent leave	−0.29	−0.95	0.41	0.46	−0.08	−1.58
Temporary ill	8.55	8.77	9.68	10.12	5.61	4.97
Retired	5.13	5.30	5.68	0.50	5.07	9.64
Unemployed	3.88	0.06	8.92	−11.77	1.42	5.29
Disabled	24.26	23.56	12.96	18.72	27.52	24.32
Underweight	1.99	−0.90	2.30	−1.02	1.40	−0.50
Overweight	−7.95	−3.90	−7.34	−2.24	−1.83	−0.10
Obese	2.63	4.98	−7.58	−1.45	7.25	11.00
Daily smoker	5.23	8.15	9.11	9.31	3.36	6.24
Weekly smoker	−0.40	−0.03	−1.50	−0.12	−0.23	−0.69
Seldom smoker	−0.77	−0.51	−0.04	−0.02	−2.48	−1.24
Former smoker	−3.91	−1.35	−4.86	−0.11	−3.17	−2.52
5+ alcoholic drinks	0.06	0.32	0.03	0.10	−0.05	−0.90

**Table 5:** Decomposition with household income as the income measure

	**Overall**		**Males**		**Females**	
	**2007**	**2009**	**2007**	**2009**	**2007**	**2009**
	**% contribution**					
**Household income**	36.23	42.33	43.58	44.18	35.06	38.79
Age	−1.30	3.21	−3.58	7.41	1.42	−1.23
Female	1.11	1.22				
Single	1.41	0.15	3.32	3.24	0.29	−4.71
In a relationship	0.19	0.22	−0.04	0.04	0.92	0.86
Cohabiting	0.13	−0.35	−0.59	1.17	0.39	1.12
Divorced	−0.01	−0.32	0.52	3.03	−1.40	−4.95
Widowed	1.53	−0.68	2.17	2.37	−0.61	−2.79
Low education	3.54	2.64	−0.94	3.04	9.07	3.56
High education	4.42	−0.12	5.14	0.23	4.32	0.01
Business owner	−0.47	0.80	−0.45	0.44	−0.11	0.61
Student	−1.84	−0.67	−7.56	−0.48	0.20	−0.53
Homemaker	0.28	1.11	0.53	0.63	−1.71	0.15
Parent leave	0.23	−0.28	0.30	0.25	−0.17	−0.32
Temporary ill	7.39	7.04	7.62	8.70	6.29	4.80
Retired	4.96	4.34	6.57	0.62	4.83	6.54
Unemployed	2.72	0.03	6.35	−3.41	1.10	1.52
Disabled	18.45	16.49	11.71	10.34	22.08	22.76
Underweight	1.20	−0.64	1.06	−0.87	1.16	−0.25
Overweight	−0.39	0.10	−0.40	0.19	1.05	1.82
Obese	4.09	5.97	−1.83	0.49	9.01	13.99
Daily smoker	7.05	7.65	7.70	6.04	6.86	9.01
Weekly smoker	−0.15	−0.01	−1.28	0.00	0.22	−0.11
Seldom smoker	−1.55	−0.53	−0.67	0.09	−2.47	−1.39
Former smoker	−0.67	0.18	−0.09	0.36	−0.98	−1.31
5+ alcoholic drinks	−0.01	0.14	−0.01	−0.15	0.05	−0.62

**Table 6:** Decomposition with equivalized household income as the income measure

	**Overall**		**Males**		**Females**	
	**2007**	**2009**	**2007**	**2009**	**2007**	**2009**
	**% contribution**					
**Equivalized household income**	28.74	41.73	25.17	44.53	36.96	41.64
Age	−0.29	2.21	−1.09	4.81	2.14	0.24
Female	1.52	1.37				
Single	1.73	0.95	3.71	2.56	0.22	−2.73
In a relationship	0.02	0.09	−0.20	−0.18	0.59	0.78
Cohabiting	−0.02	−0.07	−0.67	2.28	0.11	0.51
Divorced	0.50	0.11	1.17	2.94	−1.49	−4.56
Widowed	1.91	−0.42	2.22	2.46	−0.49	−2.13
Low education	3.64	2.75	−1.05	3.43	7.82	3.17
High education	4.20	−0.11	6.38	−0.32	3.81	0.35
Business owner	−0.41	0.60	−0.40	−0.01	−0.06	0.41
Student	−1.73	−0.45	−8.42	−0.14	0.27	−0.48
Homemaker	0.72	1.05	1.41	0.59	−2.44	0.05
Parent leave	−0.12	−2.41	−0.35	−1.08	−0.01	−3.41
Temporary ill	7.12	7.90	6.97	9.86	6.42	5.47
Retired	4.47	3.86	7.67	1.66	3.45	3.31
Unemployed	2.90	−0.01	6.79	−3.53	1.41	1.58
Disabled	19.86	14.67	13.82	9.46	22.80	19.26
Underweight	1.16	−0.57	1.28	−0.88	0.92	−0.17
Overweight	−1.17	−0.35	0.01	0.21	−0.41	0.44
Obese	4.86	7.25	−3.72	0.95	10.76	16.12
Daily smoker	6.78	7.05	8.46	5.17	6.20	8.60
Weekly smoker	−0.05	0.00	−0.98	−0.03	0.25	−0.16
Seldom smoker	−1.32	−0.39	−0.50	0.17	−1.92	−1.18
Former smoker	−1.49	−0.47	−1.77	0.18	−1.30	−2.10
5+ alcoholic drinks	0.01	0.18	0.01	−0.56	0.16	0.17

Income-related health inequality increased substantially between 2007 and 2009 for males, while it slightly decreased for females. The percentage point changes in the contribution of specific determinants to inequality between years can be seen in Table 7 for both genders and all three income measures separately. The largest increase in contributions to male inequality between years is from being a student, with a 22.96 percentage point increase in contributions when individual income was the income measure, 7.08 percentage points when household income was the income measure and 8.28 percentage points when equivalized household income was the income measure. For females being a student reduced inequality between years, but mainly when individual income was the income measure, with a 5.91 percentage point change. The contributions of low education to inequality also increased between years for males, using all income measures. For females, however, the contribution of education to inequality decreased between years and as well did the contribution of being single or divorced (specifically when household income and equivalized household income were the income measures). For males the contribution of being single increased by 4.18 percentage points between years using individual income as the income measure. The contribution of cohabiting and being divorced also increased between years for males, except for the contribution of cohabiting when individual income was the income measure.

**Table 7:** Percentage point changes in contributions to inequality between 2007 and 2009

	**Males**			**Females**		
	**Individual income**	**Household income**	**Equivalized household income**	**Individual income**	**Household income**	**Equivalized household income**
Income	−2.25	0.60	19.37	−7.26	3.73	4.68
Age	−8.52	10.99	5.90	0.87	−2.65	−1.90
Single	4.18	−0.08	−1.16	−2.36	−4.99	−2.95
In a relationship	1.78	0.08	0.02	0.46	−0.06	0.18
Cohabiting	−0.66	1.76	2.95	0.24	0.73	0.40
Divorced	0.88	2.51	1.77	1.66	−3.56	−3.07
Widowed	1.36	0.20	0.24	−1.51	−2.17	−1.64
Low education	4.94	3.98	4.48	−8.35	−5.51	−4.64
High education	−6.07	−4.91	−6.70	−6.86	−4.31	−3.46
Business owner	4.38	0.88	0.38	0.61	0.72	0.47
Student	22.96	7.08	8.28	−5.91	−0.73	−0.75
Homemaker	1.79	0.10	−0.82	1.19	1.86	2.49
Parent leave	0.05	−0.05	−0.74	−1.51	−0.16	−3.40
Temporary ill	0.44	1.08	2.90	−0.63	−1.49	−0.94
Retired	−5.18	−5.95	−6.00	4.58	1.71	−0.15
Unemployed	−20.69	−9.76	−10.33	3.87	0.42	0.17
Disability	5.76	−1.36	−4.37	−3.20	0.68	−3.54
Underweight	−3.32	−1.94	−2.16	−1.91	−1.42	−1.10
Overweight	5.10	0.59	0.20	1.74	0.77	0.85
Obese	6.13	2.31	4.66	3.76	4.98	5.36
Daily smoker	0.20	−1.67	−3.30	2.88	2.15	2.40
Weekly smoker	1.38	1.28	0.95	−0.47	−0.34	−0.41
Seldom smoker	0.02	0.76	0.66	1.24	1.08	0.75
Former smoker	4.75	0.45	1.94	0.65	−0.33	−0.80
5+ alcoholic drinks	0.07	−0.13	−0.57	−0.85	−0.67	0.01

For disabled females inequality decreased between years when individual income and equivalized household income were the income measures. For females there was an increase in the contributions of being retired or unemployed between years, but mainly when individual income was the income measure. For males the contribution of being retired decreased by around 5–6 percentage points between years as well as the contribution of being unemployed, which decreased substantially using all income measures. The largest decrease was when individual income was used, or 20.69 percentage points. Due to this decrease, as well as a decrease in contributions from other determinants that were not as substantial, the overall increase in income-related health inequality for males between years is lower than it otherwise would have been. The contributions of being a male business owner increased by 4.38 percentage points between years when individual income was the income measure and being disabled by 5.76 percentage points.

For males the contributions of being obese and overweight increased between years using all income measures, mainly when individual income was the income measure. The contribution of being obese also increased for females, as well as a minor increase in the contribution of being overweight. The contribution of being a male former smoker increased by 4.75 percentage points between years when individual income was the income measure while the contribution of smoking daily increased by 2–3 percentage points between years for females, using all income measures. For males the contribution of age increased by 10.99 percentage points when household income was the income measure and 5.90 percentage points when equivalized household income was the income measure, while it decreased slightly between years for females. The change in contribution of equivalized household income to health inequality for males between years was 19.37 percentage points. For females the contribution of individual income reduced by 7.26 percentage points between years. Other determinants, however, had a smaller effect on the change in income-related health inequality between years.

## Discussion

The results of the current study add to the existing knowledge on aggregate, as well as individual determinants of income-related disparities. Specifically this is the first study that we are aware of examining the relationship between the current economic crisis and health-income inequality. In all instances the concentration indices show that health is concentrated among those with higher income, both overall and for males and females separately, which is in line with previous studies on income-related health inequality. For both genders and in both years the health-income inequalities are largest when household income is used as the income measure in the analyses. This suggests that health inequalities do not only arise through one’s own labor-market experiences, but also though selection into marriage. For example, those who are healthy are not only likely to have relatively high income but are more likely to marry a person with high income.

The results show how income-related health inequality changes in a different manner for males and females and from the decomposition analyses several interesting differences in contributions to health inequality between genders can be seen. Health-income inequalities among females remain fairly stable between years. They slightly decrease for two of the three concentration indices, and slightly increase for one, while for males, in all instances, there is a considerable increase in health-income inequality between years. The reasons for this gender difference can only be hypothesized upon at this point. Unfortunately, as can be seen in the discussion below, there is not a single determinant of inequality that is obviously driving this change, but a mixture of factors relating to the labor-market, lifestyle and demographics. The general results are, however, partly in accordance with a Japanese study [20] where health-income inequality among young working-age people was found to increase after the Asian financial crisis of the late 1990s as well as occupational class-based inequalities in poor health were found to widen. However, they found disparities in health-income inequality to decrease among older working-age people. The current study is not in accordance with other results from Japan and Finland, where health-income inequalities were found to decrease following the Asian financial crisis [18] and the Finnish financial crisis of early 1990s [19].

In the current study the largest contributions to inequality for both genders using all three different income measures are income itself and disability. However, the magnitude of the contribution of these determinants varies considerably between genders. The increased income-related health inequality among males between years can mainly be explained by increases in contributions from being obese, having low education and being a student. After the economic collapse many individuals left the job market and started studying, but education in Iceland is mostly without tuition fees and thus substitution between unemployment and being a student may be relatively unconstrained and more likely than in societies where being a student can be seen as a considerable financial commitment. In 2009 there was for example a notable increase in new registrations at the tertiary and doctorate level [43]. There is furthermore a large change in the contribution of age and income for males between years, but these changes are sensitive to the income measure used. When examining the changes in contributions between years it can be seen that the contribution of being unemployed decreased significantly in male estimations. This shows that while the contribution of being a student reduced health inequality more in 2007 than 2009, the percentage point change in the contribution of being unemployed to inequality decreased by similar amount. Whether this is due to a substitution effect between being unemployed or a student is not clear. The same pattern can be seen for males with low education and high education, where the contribution of low education to inequality increased by similar amount as high education to inequality decreased by. There are interesting gender differences in the contribution of overweight and obesity to inequality. There is a notable change in the contribution of obesity to health-income inequality among males. However, it changes from reducing inequality to increasing it only slightly, while for females being obese contributes significantly to inequality in both years. This is in accordance with the literature on body weight and the labor market [25,44].

The purpose of the decomposition analysis was to observe if there were specific aspects of the relationship between health and income that drove cyclical variations in income-related health inequality. If such patterns were apparent, they could be focused on by policy makers. However, the overarching conclusion from the decomposition analysis is that changes in contributions to inequality are spread across demographics, labor-market determinants and lifestyle, but not overwhelmingly to be found within one subgroup of variables.

The current study has both strengths and limitations. The short time frame of the study period is a strength and the data give an opportunity to study income-related inequality in health before and after a deep and sharp economic collapse. Due to the speed of change, this economic collapse has already proved to be an important tool in examining the effects of business cycles [24,25,45]. Only individuals who answered both waves are used in the analyses, which is especially important due to the fact that the data used come from a subjective self-report questionnaire. As the survey used is very rich in variables, a detailed decomposition analysis could be done and three different income measures employed. However, while self-assessed health has proven to be a good and valid indicator of health, there has been some concern about to what degree self-assessed measures are comparable across socio-economic groups. These measures may suffer from a heterogeneous reporting bias since different people might not rate health in the same way, and thus income-related health inequality might be biased [46,47]. While this is a concern it should be noted that time-invariant individual reporting heterogeneity should not be a problem as the analysis is done on a balanced panel.

This study provides evidence on how income-related inequality in health changed after the Icelandic economic collapse in 2008 and shows an increase in health-income inequality, mainly among males. This is only the first step in understanding this relationship between health distribution and income and what determinants are the largest contributors to inequality. Further research is needed before a clear picture of this relationship emerges. As is done here, it may be sensible to use a broad measure of health when examining the effect of the Icelandic economic collapse for the first time. However, given that those results indicate that changes in the income-related distribution of health occurred, a logical next step would be to examine this relationship using other and more specific measures of health, as well as conducting examinations in other countries and using different data.

## Competing interests

The authors declare that they have no competing interests.

## Authors’ contributions

Both authors collaborated on all tasks and sections of the paper. Both authors read and approved the final manuscript.

## Supplementary Material

Additional file 1: Table S1 The relationship between SAH and determinants, using individual income. **Table S2.** The relationship between SAH and determinants, using household income. **Table S3.** The relationship between SAH and determinants, using equivalized household income. Click here for file
