# Supplementary material for: Health-income inequality: the effects of the Icelandic economic collapse
Source: Int J Equity Health. 2014 Jul 25;13:50. doi: 10.1186/1475-9276-13-50 (PMC4119249; doi:10.1186/1475-9276-13-50)
Supplement: Additional file 1: Table S1 — The relationship between SAH and determinants, using individual income. Table S2. The relationship between SAH and determinants, using household income. Table S3. The relationship between SAH and determinants, using equivalized household income. [file 1475-9276-13-50-S1.doc]

**Additional file 1**

**Table S**1 The relationship between SAH and determinants, using individual income

| **Dependent variable:** | **Males** | | | | | | **Females** | | | | | |
| --- | --- | --- | --- | --- | --- | --- | --- | --- | --- | --- | --- | --- |
| **Self-assessed health** | **2007** | | | **2009** | | | **2007** | | | **2009** | | |
| **(SAH)** | **Coefficient** | **Robust SE** | **p value** | **Coefficient** | **Robust SE** | **p value** | **Coefficient** | **Robust SE** | **p value** | **Coefficient** | **Robust SE** | **p value** |
| **Variable:** | | | | | | | | | | | | |
| Individual income | 0.023** | 0.011 | 0.039 | 0.033** | 0.015 | 0.025 | 0.022** | 0.010 | 0.037 | 0.019 | 0.016 | 0.218 |
| Age | 0.002 | 0.003 | 0.519 | −0.007** | 0.003 | 0.028 | −0.002 | 0.002 | 0.400 | 0.000 | 0.002 | 0.917 |
| Single | −0.086 | 0.089 | 0.335 | −0.152 | 0.113 | 0.178 | −0.079 | 0.073 | 0.279 | 0.042 | 0.079 | 0.595 |
| In a relationship | 0.019 | 0.132 | 0.886 | −0.018 | 0.142 | 0.897 | −0.175** | 0.089 | 0.048 | −0.112 | 0.108 | 0.297 |
| Cohabiting | 0.038 | 0.074 | 0.610 | −0.171* | 0.090 | 0.056 | −0.012 | 0.061 | 0.844 | 0.023 | 0.062 | 0.713 |
| Divorced | −0.063 | 0.117 | 0.590 | −0.213 | 0.148 | 0.152 | −0.005 | 0.082 | 0.954 | 0.116* | 0.069 | 0.090 |
| Widowed | −0.284 | 0.174 | 0.104 | −0.388* | 0.216 | 0.073 | −0.074 | 0.093 | 0.432 | 0.065 | 0.103 | 0.526 |
| Low education | −0.008 | 0.064 | 0.899 | −0.093 | 0.071 | 0.192 | −0.146** | 0.058 | 0.012 | −0.042 | 0.061 | 0.487 |
| High education | 0.045 | 0.062 | 0.469 | −0.005 | 0.068 | 0.936 | 0.046 | 0.053 | 0.383 | −0.013 | 0.054 | 0.816 |
| Business owner | −0.028 | 0.058 | 0.628 | 0.057 | 0.059 | 0.332 | −0.063 | 0.082 | 0.443 | 0.106 | 0.089 | 0.233 |
| Student | 0.266*** | 0.096 | 0.006 | 0.107 | 0.108 | 0.324 | −0.010 | 0.064 | 0.880 | 0.054 | 0.065 | 0.403 |
| Homemaker | 0.016 | 0.123 | 0.897 | −0.080 | 0.100 | 0.427 | 0.047 | 0.070 | 0.503 | 0.031 | 0.075 | 0.674 |
| Parent leave | 0.067 | 0.220 | 0.762 | 0.130 | 0.181 | 0.472 | 0.008 | 0.109 | 0.944 | 0.156 | 0.115 | 0.173 |
| Temporary ill | −0.494*** | 0.174 | 0.005 | −0.672*** | 0.252 | 0.008 | −0.415*** | 0.136 | 0.002 | −0.606*** | 0.135 | 0.000 |
| Retired | −0.102 | 0.087 | 0.237 | −0.009 | 0.100 | 0.931 | −0.130 | 0.093 | 0.163 | −0.151 | 0.107 | 0.158 |
| Unemployed | −0.399** | 0.187 | 0.032 | 0.179 | 0.153 | 0.243 | −0.089 | 0.112 | 0.430 | −0.186* | 0.108 | 0.086 |
| Disability | −0.341* | 0.187 | 0.069 | −0.563*** | 0.140 | 0.000 | −0.783*** | 0.119 | 0.000 | −0.669*** | 0.126 | 0.000 |
| Underweight | −0.806 | 0.502 | 0.109 | 0.440 | 0.478 | 0.358 | −0.542 | 0.438 | 0.216 | 0.246 | 0.161 | 0.128 |
| Overweight | −0.114* | 0.060 | 0.058 | −0.064 | 0.073 | 0.377 | −0.155*** | 0.046 | 0.001 | −0.179*** | 0.049 | 0.000 |
| Obese | −0.388*** | 0.079 | 0.000 | −0.323*** | 0.088 | 0.000 | −0.470*** | 0.056 | 0.000 | −0.465*** | 0.058 | 0.000 |
| Daily smoker | −0.262*** | 0.086 | 0.002 | −0.264** | 0.110 | 0.016 | −0.259*** | 0.067 | 0.000 | −0.258*** | 0.063 | 0.000 |
| Weekly smoker | −0.192 | 0.235 | 0.414 | 0.057 | 0.181 | 0.755 | −0.067 | 0.152 | 0.662 | −0.141 | 0.176 | 0.424 |
| Seldom smoker | −0.058 | 0.150 | 0.699 | −0.004 | 0.176 | 0.981 | −0.239* | 0.132 | 0.072 | −0.308** | 0.152 | 0.043 |
| Former smoker | −0.118* | 0.063 | 0.061 | −0.024 | 0.064 | 0.709 | −0.098** | 0.049 | 0.047 | −0.111** | 0.053 | 0.038 |
| 5+ alcoholic drinks | 0.000 | 0.001 | 0.918 | 0.000 | 0.001 | 0.920 | 0.001 | 0.001 | 0.283 | 0.003*** | 0.001 | 0.009 |
| Number of obs | 1234 | | | 949 | | | 1355 | | | 1102 | | |
| R-squared | 0.147 | | | 0.160 | | | 0.246 | | | 0.240 | | |

*p < 0.10 **p < 0.05 ***p < 0.01.

**Table S2** The relationship between SAH and determinants, using household income

| **Dependent variable:** | **Males** | | | | | | **Female** | | | | | |
| --- | --- | --- | --- | --- | --- | --- | --- | --- | --- | --- | --- | --- |
| **Self-assessed health** | **2007** | | | **2009** | | | **2007** | | | **2009** | | |
| **(SAH)** | **Coefficient** | **Robust SE** | **p value** | **Coefficient** | **Robust SE** | **p value** | **Coefficient** | **Robust SE** | **p value** | **Coefficient** | **Robust SE** | **p value** |
| **Variable:** | | | | | | | | | | | | |
| Household income | 0.012** | 0.006 | 0.027 | 0.021*** | 0.008 | 0.007 | 0.014*** | 0.005 | 0.003 | 0.019*** | 0.007 | 0.005 |
| Age | 0.003 | 0.003 | 0.254 | −0.006** | 0.003 | 0.047 | −0.001 | 0.002 | 0.713 | 0.001 | 0.002 | 0.756 |
| Single | −0.068 | 0.090 | 0.450 | −0.116 | 0.115 | 0.312 | −0.008 | 0.077 | 0.920 | 0.119 | 0.084 | 0.155 |
| In a relationship | −0.030 | 0.134 | 0.819 | −0.010 | 0.148 | 0.946 | −0.133 | 0.093 | 0.152 | −0.074 | 0.111 | 0.505 |
| Cohabiting | 0.055 | 0.076 | 0.464 | −0.138 | 0.092 | 0.134 | 0.022 | 0.063 | 0.725 | 0.041 | 0.064 | 0.518 |
| Divorced | −0.030 | 0.125 | 0.809 | −0.218 | 0.155 | 0.158 | 0.054 | 0.090 | 0.550 | 0.188** | 0.073 | 0.010 |
| Widowed | −0.283 | 0.196 | 0.148 | −0.324 | 0.220 | 0.142 | 0.025 | 0.097 | 0.792 | 0.100 | 0.111 | 0.367 |
| Low education | 0.019 | 0.066 | 0.774 | −0.072 | 0.072 | 0.316 | −0.130** | 0.060 | 0.030 | −0.047 | 0.063 | 0.458 |
| High education | 0.070 | 0.061 | 0.246 | 0.003 | 0.066 | 0.964 | 0.072 | 0.053 | 0.176 | 0.000 | 0.054 | 0.998 |
| business owner | −0.027 | 0.058 | 0.642 | 0.061 | 0.058 | 0.296 | −0.051 | 0.083 | 0.537 | 0.116 | 0.093 | 0.213 |
| Student | 0.249*** | 0.093 | 0.008 | 0.034 | 0.106 | 0.747 | −0.026 | 0.062 | 0.679 | 0.042 | 0.064 | 0.514 |
| Homemaker | −0.028 | 0.126 | 0.821 | −0.055 | 0.103 | 0.591 | 0.048 | 0.072 | 0.503 | −0.005 | 0.077 | 0.951 |
| Parent leave | 0.076 | 0.225 | 0.735 | 0.141 | 0.185 | 0.446 | −0.015 | 0.114 | 0.893 | 0.208* | 0.120 | 0.084 |
| Temporary ill | −0.450** | 0.180 | 0.012 | −0.801*** | 0.259 | 0.002 | −0.506*** | 0.141 | 0.000 | −0.545*** | 0.139 | 0.000 |
| Retired | −0.138 | 0.088 | 0.115 | −0.012 | 0.101 | 0.907 | −0.114 | 0.099 | 0.250 | −0.101 | 0.113 | 0.371 |
| Unemployed | −0.405** | 0.195 | 0.038 | 0.145 | 0.164 | 0.377 | −0.128 | 0.104 | 0.219 | −0.131 | 0.113 | 0.247 |
| Disability | −0.399* | 0.204 | 0.051 | −0.585*** | 0.137 | 0.000 | −0.717*** | 0.130 | 0.000 | −0.672*** | 0.130 | 0.000 |
| Underweight | −0.818* | 0.494 | 0.098 | 0.486 | 0.515 | 0.345 | −0.469 | 0.423 | 0.268 | 0.251 | 0.159 | 0.114 |
| Overweight | −0.114* | 0.060 | 0.058 | −0.042 | 0.076 | 0.581 | −0.155*** | 0.048 | 0.001 | −0.162*** | 0.051 | 0.002 |
| Obese | −0.378*** | 0.080 | 0.000 | −0.287*** | 0.092 | 0.002 | −0.483*** | 0.058 | 0.000 | −0.473*** | 0.060 | 0.000 |
| Daily smoker | −0.241*** | 0.091 | 0.008 | −0.253** | 0.113 | 0.026 | −0.255*** | 0.069 | 0.000 | −0.258*** | 0.064 | 0.000 |
| Weekly smoker | −0.212 | 0.239 | 0.375 | 0.033 | 0.181 | 0.854 | −0.097 | 0.152 | 0.524 | −0.165 | 0.174 | 0.345 |
| Seldom smoker | −0.069 | 0.162 | 0.667 | 0.018 | 0.175 | 0.918 | −0.239* | 0.133 | 0.072 | −0.325 | 0.155 | 0.037 |
| Former smoker | −0.119* | 0.064 | 0.062 | −0.023 | 0.064 | 0.716 | −0.112** | 0.051 | 0.029 | −0.123 | 0.055 | 0.026 |
| 5+ alcoholic drinks | 0.000 | 0.001 | 0.899 | −0.001 | 0.001 | 0.586 | 0.001 | 0.001 | 0.375 | 0.003 | 0.001 | 0.007 |
| Number of obs | 1202 | | | 927 | | | 1283 | | | 1044 | | |
| R-squared | 0.144 | | | 0.160 | | | 0.243 | | | 0.245 | | |

*p < 0.10 **p < 0.05 ***p < 0.01.

**Table S3** The relationship between SAH and determinants, using equivalized household income

| **Dependent variable:** | **Males** | | | | | | **Females** | | | | | |
| --- | --- | --- | --- | --- | --- | --- | --- | --- | --- | --- | --- | --- |
| **Self-assessed health** | **2007** | | | **2009** | | | **2007** | | | **2009** | | |
| **(SAH)** | **Coefficient** | **Robust SE** | **p value** | **Coefficient** | **Robust SE** | **p value** | **Coefficient** | **Robust SE** | **p value** | **Coefficient** | **Robust SE** | **p value** |
| **Variable:** | | | | | | | | | | | | |
| Equivalized hh income | 0.007 | 0.007 | 0.317 | 0.022*** | 0.008 | 0.008 | 0.017*** | 0.005 | 0.001 | 0.024*** | 0.008 | 0.004 |
| Age | 0.003 | 0.003 | 0.264 | −0.007** | 0.003 | 0.032 | −0.002 | 0.002 | 0.378 | 0.000 | 0.002 | 0.898 |
| Single | −0.105 | 0.088 | 0.231 | −0.163 | 0.114 | 0.153 | −0.007 | 0.074 | 0.922 | 0.088 | 0.082 | 0.283 |
| In a relationship | −0.054 | 0.132 | 0.681 | −0.041 | 0.147 | 0.778 | −0.153 | 0.093 | 0.102 | −0.099 | 0.110 | 0.370 |
| Cohabiting | 0.028 | 0.077 | 0.717 | −0.147 | 0.091 | 0.107 | 0.008 | 0.064 | 0.901 | 0.025 | 0.064 | 0.695 |
| Divorced | −0.071 | 0.124 | 0.566 | −0.246 | 0.154 | 0.110 | 0.058 | 0.091 | 0.522 | 0.184** | 0.073 | 0.012 |
| Widowed | −0.299 | 0.205 | 0.146 | −0.332 | 0.224 | 0.138 | 0.021 | 0.097 | 0.832 | 0.084 | 0.111 | 0.446 |
| Low education | 0.018 | 0.066 | 0.785 | −0.078 | 0.072 | 0.280 | −0.134** | 0.060 | 0.026 | −0.052 | 0.063 | 0.415 |
| High education | 0.078 | 0.061 | 0.203 | −0.004 | 0.065 | 0.945 | 0.075 | 0.053 | 0.156 | 0.005 | 0.054 | 0.928 |
| Business owner | −0.022 | 0.059 | 0.712 | 0.075 | 0.059 | 0.199 | −0.043 | 0.083 | 0.605 | 0.128 | 0.094 | 0.171 |
| Student | 0.236** | 0.093 | 0.011 | 0.014 | 0.106 | 0.892 | −0.041 | 0.061 | 0.502 | 0.035 | 0.064 | 0.585 |
| Homemaker | −0.070 | 0.134 | 0.603 | −0.053 | 0.105 | 0.611 | 0.060 | 0.073 | 0.413 | −0.001 | 0.079 | 0.986 |
| Parent leave | 0.099 | 0.224 | 0.658 | 0.172 | 0.184 | 0.351 | −0.005 | 0.114 | 0.965 | 0.222* | 0.119 | 0.064 |
| Temporary ill | −0.451** | 0.187 | 0.016 | −0.800*** | 0.257 | 0.002 | −0.542*** | 0.145 | 0.000 | −0.538*** | 0.138 | 0.000 |
| Retired | −0.150 | 0.092 | 0.102 | −0.034 | 0.106 | 0.752 | −0.083 | 0.101 | 0.412 | −0.053 | 0.117 | 0.648 |
| Unemployed | −0.407** | 0.198 | 0.041 | 0.158 | 0.168 | 0.347 | −0.154 | 0.109 | 0.156 | −0.131 | 0.113 | 0.247 |
| Disabled | −0.420** | 0.207 | 0.042 | −0.598*** | 0.140 | 0.000 | −0.730*** | 0.135 | 0.000 | −0.670*** | 0.130 | 0.000 |
| Underweight | −0.840* | 0.497 | 0.091 | 0.484 | 0.525 | 0.357 | −0.473 | 0.429 | 0.271 | 0.248 | 0.156 | 0.112 |
| Overweight | −0.127** | 0.061 | 0.037 | −0.050 | 0.076 | 0.507 | −0.157*** | 0.048 | 0.001 | −0.166*** | 0.051 | 0.001 |
| Obese | −0.387*** | 0.081 | 0.000 | −0.292*** | 0.092 | 0.002 | −0.465*** | 0.058 | 0.000 | −0.475*** | 0.061 | 0.000 |
| Daily smoker | −0.256*** | 0.090 | 0.005 | −0.259** | 0.113 | 0.022 | −0.254*** | 0.070 | 0.000 | −0.255*** | 0.064 | 0.000 |
| Weekly smoker | −0.197 | 0.239 | 0.410 | 0.028 | 0.181 | 0.876 | −0.104 | 0.152 | 0.496 | −0.162 | 0.178 | 0.364 |
| Seldom smoker | −0.045 | 0.164 | 0.785 | 0.042 | 0.176 | 0.810 | −0.243* | 0.133 | 0.069 | −0.331** | 0.157 | 0.036 |
| Former smoker | −0.126* | 0.065 | 0.051 | −0.028 | 0.064 | 0.662 | −0.118** | 0.052 | 0.022 | −0.122** | 0.056 | 0.028 |
| 5+ alcoholic drinks | 0.000 | 0.001 | 0.992 | −0.001 | 0.001 | 0.463 | 0.001 | 0.001 | 0.472 | 0.003*** | 0.001 | 0.007 |
| Number of obs | 1168 | | | 904 | | | 1255 | | | 1032 | | |
| R-squared | 0.140 | | | 0.161 | | | 0.241 | | | 0.246 | | |

*p < 0.10 **p < 0.05 ***p < 0.01.
